# Phylogenomic Classification and Biosynthetic Potential of the Fossil Fuel-Biodesulfurizing *Rhodococcus* Strain IGTS8

**DOI:** 10.3389/fmicb.2020.01417

**Published:** 2020-07-07

**Authors:** Dean Thompson, Valérie Cognat, Michael Goodfellow, Sandrine Koechler, Dimitri Heintz, Christine Carapito, Alain Van Dorsselaer, Huda Mahmoud, Vartul Sangal, Wael Ismail

**Affiliations:** ^1^Faculty of Health and Life Sciences, Northumbria University, Newcastle upon Tyne, United Kingdom; ^2^Institut de Biologie Moléculaire des Plantes, Centre National de Recherche Scientifique (CNRS), Université de Strasbourg, Strasbourg, France; ^3^School of Natural and Environmental Sciences, Newcastle University, Newcastle upon Tyne, United Kingdom; ^4^Laboratoire de Spectrométrie de Masse Bio-organique, Institut Pluridisciplinaire Hubert Curien, UMR 7178 CNRS, Université de Strasbourg, Strasbourg, France; ^5^Department of Biological Sciences, College of Science, Kuwait University, Safat, Kuwait; ^6^Environmental Biotechnology Program, Life Sciences Department, College of Graduate Studies, Arabian Gulf University, Manama, Bahrain

**Keywords:** biodesulfurization, *Rhodococcus*, 4S pathway, phylogenomics, dibenzothiophene, average nucleotide identity, digital DNA-DNA hybridization

## Abstract

*Rhodococcus* strain IGTS8 is the most extensively studied model bacterium for biodesulfurization of fossil fuels via the non–destructive sulfur–specific 4S pathway. This strain was initially assigned to *Rhodococcus rhodochrous* and later to *Rhodococcus erythropolis* thus making its taxonomic status debatable and reflecting the limited resolution of methods available at the time. In this study, phylogenomic analyses of the whole genome sequences of strain IGTS8 and closely related rhodococci showed that *R. erythropolis* and *Rhodococcus qingshengii* are very closely related species, that *Rhodococcus* strain IGTS8 is a *R. qingshengii* strain and that several strains identified as *R. erythropolis* should be re-classified as *R. qingshengii*. The genomes of strains assigned to these species contain potentially novel biosynthetic gene clusters showing that members of these taxa should be given greater importance in the search for new antimicrobials and other industrially important biomolecules. The plasmid-borne *dsz* operon encoding fossil fuel desulfurization enzymes was present in *R. qingshengii* IGTS8 and *R. erythropolis* XP suggesting that it might be transferable between members of these species.

## Introduction

The implementation of green and economic technologies in the petroleum industry is of increasing interest. This is particularly important in view of the continuously increasing global demand for cleaner fuels, stricter environmental regulations and dwindling oil prices (Sahu et al., 2015; Head and Gray, 2016; Ismail et al., 2017). The biotechnological desulfurization of crude oil and transportation fuels, such as diesel and gasoline, holds the potential to overcome or mitigate the technical, economic and environmental hurdles of conventional thermochemical desulfurization technologies (Kilbane, 2006; Mohebali and Ball, 2016). *Rhodococcus* strain IGTS8 (ATCC 53968) was the first bacterium to be isolated based on its unique ability to break C-S bonds of fuel-born organosulfur compounds and utilize them as a sole sulfur source (Denome et al., 1994). This discovery raised the prospect of using biocatalysts for removal of sulfur from fossil fuels via biodesulfurization (Kilbane and Bielaga, 1990; Kilbane II and Jackowski, 1992).

The biodesulfurization reactions are catalyzed by two monooxygenases (DszC and DszA) and a desulfinase (DszB) which constitute the non-destructive 4S pathway. This pathway was first reported and characterized in the IGTS8 strain which for many years has served as the most commonly adopted model for biodesulfurization studies. The genes of the 4S pathway, *dszA*, *dszB*, and *dszC*, are carried as an operon on a linear megaplasmid in the IGTS8 strain (Kilbane and Stark, 2016). These genes have been found in *R. erythropolis* strains A66 and A69 which use various organosulfur and organonitrogen compounds as sole sources of sulfur and nitrogen, respectively (Santos et al., 2007). DszC shows sulfur acquisition oxidoreductase (SfnB family) and acyl-CoA dehydrogenase activities and catalyzes the conversion of dibenzothiophene to dibenzothiophene sulfone (Gray et al., 1996). DszA, a FMN-dependent oxidoreductase of the nitrilotriacetate monooxygenase family, converts dibenzothiophene sulfone to 2-(2′-hydroxyphenyl) benzenesulfinate which is catalytically converted to 2-hydroxybiphenyl and sulfite by the desulfinase DszB.

*Rhodococcus* strain IGTS8 was classified as *Rhodococcus rhodochrous* (Zopf, 1891; Tsukamura, 1974) by Kayser et al. (1993) prior to being reclassified as *R. erythropolis* (Gray and Thornton, 1928; Goodfellow and Alderson, 1977; Oldfield et al., 1998; Santos et al., 2007). These initial classifications likely reflect the limited resolution of the taxonomic methods available at the time (Goodfellow et al., 1998, 1999). Members of the genus *Rhodococcus* (Zopf, 1891) emend Goodfellow et al. (1998) are known for their metabolic versatility and potential biotechnological applications in areas such as bioremediation, biotransformation and biocatalysis. They also produce biosurfactants, carotenoids and bioflocculation agents of industrial interest (Gürtler and Seviour, 2010; Jones and Goodfellow, 2012).

The extent of the heterogeneity encompassed within the genus *Rhodococcus* became apparent as a result of the application of phylogenomic methods (Sangal et al., 2016, 2019). Such studies have benefited from the use of improved metrics for the definition of species and genera (Chun and Rainey, 2014; Qin et al., 2014; Chun et al., 2018; Sant’Anna et al., 2019). Sangal and his colleagues assigned representative rhodococci to multiple well circumscribed species-groups named after the species that held priority (Sangal et al., 2016, 2019). The *R. erythropolis* species-group included the type strains *R. erythropolis, R. enclensis* (Dastager et al., 2014), *R. globerulus* (Goodfellow et al., 1982) and *R. qingshengii* (Xu et al., 2007; Táncsics et al., 2014).

The importance of resolving the taxonomic status of rhodococci which show remarkable catabolic activity is often overlooked despite significant improvements in the classification of the genus *Rhodococcus*. In the present study, the whole genome sequence of *Rhodococcus* strain IGTS8 was compared with those of *R. erythropolis* and *R. qingshengii* strains to establish its taxonomic status and to compare the biosynthetic and biodesulfurization capabilities of strains assigned to these species.

## Materials and Methods

### Bacterial Strain

*Rhodococcus* strain IGTS8 (American Type Culture Collection, ATCC 53968) was isolated by Kilbane and colleagues at the Gas Technology Institute-Chicago based on its sulfur-specific fuel biodesulfurization capabilities (Kilbane and Bielaga, 1990; Kilbane II and Jackowski, 1992).

### Genomic DNA Extraction and Sequencing

*Rhodococcus* strain IGTS8 was grown overnight in nutrient broth at 28°C and the cells pelleted by centrifugation. Genomic DNA was extracted with the Wizard Genomic DNA purification kit (Promega) using the manufacturer’s instructions for Gram positive bacteria and a library constructed with the Nextera XT DNA Library Prep kit (Ilumina) using 1 ng of genomic DNA. The library was evaluated with a 2100 Bioanalyzer (Agilent Technologies) before sequencing on an Illumina Miseq system (2 × 150 paired end reads).

### Genome Assembly and Computational Analyses

The PCR duplicates in the libraries were filtered with FastUniq 1.1 (Xu et al., 2012) and low-quality (<30) and adapter sequences trimmed with cutadapt 1.14 (Martin, 2011). Contaminant screening was performed with FastQ Screen 0.11.1 (Wingett and Andrews, 2018) to filter reads mapping on the PhiX sequence, vector sequences in the NCBI UniVec database^1^ and other species including *Homo sapiens* (hg19), *Arabidopsis thaliana* (TAIR10), *Escherichia coli* (NC_000913.3), *Stenotrophomonas maltophilia* (NC_010943.1) and *Pseudomonas aeruginosa* (NC_002516.2). The remaining singleton reads in the data were excluded with the tool makepairs of the Pairfq algorithm^2^ and the quality of the data checked with FastQC^3^. The genome sequence was assembled with SPAdes 3.10.1 (Bankevich et al., 2012) using kmer values of 25, 35, 45, 55, 65, 85, 105 and 125 in the gage mode. The quality of the assembly was assessed with QUAST 4.5 (Gurevich et al., 2013). A 16S rRNA gene sequence extracted from strain IGTS8 using BARRNAP 0.8 (Seemann, 2013) was BLAST-searched into the GenBank (Camacho et al., 2009). An extended scaffold was created from the reference genome of *Rhodococcus qingshengii* strain djl6-2 using the software Ragout 2.0 (Kolmogorov et al., 2014) and the resulting assembly annotated using the RAST-SEED pipeline (Aziz et al., 2008).

The genome sequences of other *R. qingshengii* strains and those of the closely related *Rhodococcus erythropolis* were obtained from the GenBank (Table 1). These genomes were also annotated using the RAST pipeline in order to have an equivalence of annotation for the comparative analyses. The genome sequences were compared using Roary (Page et al., 2015). A phylogenetic tree was calculated from concatenated nucleotide sequences of the core genome (1985 genes) after removing the sites with gaps using IQ-Tree (Nguyen et al., 2015). Pairwise digital DNA-DNA hybridization (dDDH) values were calculated using GGDC 2.1 (Auch et al., 2010) and average nucleotide identity (ANI) values determined using FastANI (Jain et al., 2018) between the strains within the dataset. Average amino acid identities (AAI) from reciprocal best hits were calculated between *R. erythropolis* NBRC 15567^T^, *R. qingshengii* JCM 15477^T^ and the IGTS8 strain using the web server (Rodriguez-Rivera and Konstantinidis, 2014)^4^.

**TABLE 1:** List of strains analyzed in this study.

Species	Strain ID	Assembly accession	Assembly	Size (Mb)	GC (mol%)	Designation changed
*R. erythropolis*	ACN1	ASM230387v1	Draft	7.24	62.3	
*R. erythropolis*	AV96	ASM223371v1	Draft	6.44	62.4	
*R. erythropolis*	1159	ASM209193v1	Draft	7.09	62.3	
*R. erythropolis*	IEGM 267	ASM190074v1	Draft	7.18	62.3	*R. qingshengii*
*R. erythropolis*	VSD3	ASM183130v1	Draft	6.55	62.4	
*R. erythropolis*	MI2	ASM176688v1	Draft	7.18	62.3	
*R. erythropolis*	NSX2	ASM171584v1	Draft	6.28	62.4	
*R. erythropolis*	NBRC 15567T	ASM155259v1	Draft	6.59	62.4	
*R. erythropolis*	R138	ASM69667v2	Complete	6.81	62.3	
*R. erythropolis*	JCM 9803	ASM131272v1	Draft	6.87	62.3	*R. qingshengii*
*R. erythropolis*	JCM 9805	ASM131274v1	Draft	6.96	62.4	*R. qingshengii*
*R. erythropolis*	JCM 9804	ASM131324v1	Draft	6.55	62.4	
*R. erythropolis*	CAS922i	ASM102022v1	Draft	7.20	62.3	*R. qingshengii*
*R. erythropolis*	BG43	ASM97517v1	Complete	6.87	62.3	
*R. erythropolis*	S-43	ASM83035v1	Draft	6.81	62.2	
*R. erythropolis*	JCM6824	ASM74774v1	Draft	7.02	62.3	*R. qingshengii*
*R. erythropolis*	NRRL B-16532	ASM71998v1	Draft	6.94	62.4	
*R. erythropolis*	DN1	GCF_000454425.1	Draft	6.55	62.4	
*R. erythropolis*	CCM2595	ASM45404v1	Complete	6.37	62.5	
*R. erythropolis*	XP	ASM22566v2	Draft	7.23	62.3	
*R. erythropolis*	SK121	ASM17483v1	Draft	6.79	62.5	*R. qingshengii*
*R. erythropolis*	PR4	ASM1010v1	Complete	6.90	62.3	
*R. qingshengii*	djl-6-2	ASM289396v1	Complete	6.70	62.4	
*R. qingshengii*	MK1	ASM208702v1	Draft	6.47	62.5	
*R. qingshengii*	CS98	ASM166250v1	Draft	6.71	62.4	
*R. qingshengii*	JCM 15477T	ASM164674v1	Draft	7.26	62.4	
*R. qingshengii*	CW25	ASM162343v1	Draft	6.40	62.5	
*R. qingshengii*	TUHH-12	ASM69845v1	Draft	7.43	61.7	
*R. qingshengii*	BKS 20-40	GCA_000341815.1	Draft	6.60	62.4	
*R. erythropolis*	IGTS8	GCA_006384225.1	Complete	7.01	62.4	*R. qingshengii*

### Biosynthetic and Biodesulfurization Potential

Whole genome sequences of all of the strains were analyzed using antiSMASH v5.1.1 to identify biosynthetic gene clusters (BGCs) using “Strict” detection criteria and additional features (KnownClusterBlast, ClusterBlast, SubClusterBlast, ActiveSiteFinder and Cluster Pfam analysis; Blin et al., 2019). The BGCs from the strains were sorted into groups based on their predicted activity. The protein sequences of the biodesulfurization enzymes DszA, DszB and DszC were obtained from the GenBank (accession number: L37363) and sought within the dataset using BLASTP (Camacho et al., 2009), as mentioned above.

## Results and Discussion

### Genomic Features

The sequencing reads for strain IGTS8 were assembled into 25 contigs (Table 2). The largest scaffold was 6.4 Mb in size (digital GC content 61.7 mol%) and was annotated with 6,132 coding DNA sequences, 2 rRNA and 54 tRNA genes. Other smaller contigs were potential plasmid sequences that varied from 1.2 to 88.4 kb in size and contained 0–94 genes (Table 2). The genome assembly of the IGTS8 strain was submitted to GenBank under the accession numbers CP029297–CP029321.

**TABLE 2:** Genomic features of *R. qingshengii* strain IGTS8.

Contigs	Accession number	Size (bp)	GC (mol%)	#CDS	rRNA genes	tRNA
IGTS8_Scaffold	CP029297	6442598	61.72	6132	2	54
IGTS8_Plasmid1	CP029298	42410	63.23	42		
NODE_19_Plasmid	CP029299	88412	61.84	94		
NODE_21_Plasmid	CP029300	64469	61.99	83		
NODE_22_Plasmid	CP029301	61977	61.54	60		
NODE_23_Plasmid	CP029302	17249	58.51	19		
NODE_25_Plasmid	CP029303	56340	60.91	62		
NODE_28_Plasmid	CP029304	40531	62.88	48		
NODE_29_Plasmid	CP029305	32351	57.63	27		
NODE_30_Plasmid	CP029306	31520	58.84	29		
NODE_32_Plasmid	CP029307	28097	62.96	33		
NODE_34_Plasmid	CP029308	21657	60.14	20		
NODE_37_Plasmid	CP029309	15574	62.17	15		
NODE_38_Plasmid	CP029310	14699	60.84	21		
NODE_39_Plasmid	CP029311	12554	60.84	11	1	
NODE_40_Plasmid	CP029312	8513	62.7	9		
NODE_41_Plasmid	CP029313	8462	58	6		
NODE_45_Plasmid	CP029314	5482	59.61	8		
NODE_46_Plasmid	CP029315	5051	59.55	7		
NODE_47_Plasmid	CP029316	4941	55.47		3	
NODE_50_Plasmid	CP029317	1757	64.88	2		
NODE_52_Plasmid	CP029318	1571	60.73			
NODE_54_Plasmid	CP029319	1462	62.04	1		
NODE_56_Plasmid	CP029320	1327	63.07			
NODE_59_Plasmid	CP029321	1181	63.93	1		

### Phylogenomic Characterization

A BLAST search of an extracted 16S rRNA gene sequence from the genome of *Rhodococcus* strain IGTS8 showed 99.92% identity to *R. erythropolis* strain X5 and *R. qingshengii* strain RL1. Consequently, we downloaded the genome sequences of 29 *R. erythropolis* and *R. qingshengii* strains available from GenBank and calculated dDDH values against strain IGTS8 (Table 3). The dDDH value between the latter and *R. erythropolis* NBRC 15567^T^ was 62.1% which is below the accepted species cut-off of 70% (Auch et al., 2010). In contrast, the dDDH value between strain IGTS8 and *R. qingshengii* JCM 15477^T^ was 98.2%, hence well above this threshold. The corresponding ANI values between strain IGTS8 and the type strains of *R. erythropolis* and *R. qingshengii* were 95.48 and 99.68, respectively, that is slightly and well above the 95% recognized threshold for the delineation of species (Konstantinidis and Tiedje, 2005). Similarly, the AAI value between strain IGTS8 and *R. qingshengii* JCM 15477^T^ (99.31%; SD: 5.07%) was much higher than that calculated against *R. erythropolis* NBRC 15567^T^ (96.67%; SD: 7.09%). In addition, partial sequences of the *catA* and *gyrB* genes of *R. qingshengii* strain djl-6^T^ (= DSM 45222^T^ = JCM 15477^T^) were obtained from the GenBank (accession numbers KF500432.1 and KF374699.1, respectively). BLAST search (Altschul et al., 1997; Camacho et al., 2009) for these genes not only gave single hits in the genome of strain IGTS8 but also showed 100% identity in each case. These results indicate that strain IGTS8 is more closely related to the *R. qingshengii* strain than to the type strain of *R. erythropolis*.

**TABLE 3:** dDDH values (recommended formula #2), ANI values, GGDC distances and differences in GC content between the genomes of *Rhodococcus* strain IGTS8 and *R. erythropolis* and *R. qingshengii* strains obtained from GenBank.

Strain	dDDH	FAST-ANI	GGDC distance	GC difference
*R. qingshengii* JCM 15477^T^	98.2	99.68	0.0028	0.01
*R. qingshengii* CW25	90.2	98.86	0.0119	0.14
*R. erythropolis* IEGM 267	88.4	98.55	0.0139	0.06
*R. erythropolis* JCM 9803	83.6	98.07	0.0192	0.04
*R. erythropolis* JCM 9805	87.5	98.53	0.0148	0.01
*R. erythropolis* JCM 9804	83.7	98.09	0.0191	0.06
*R. erythropolis* CAS922i	88.4	98.65	0.0139	0.05
*R. erythropolis* JCM6824	88.6	98.66	0.0137	0.08
*R. erythropolis* NRRL B-16532	80.5	97.81	0.0228	0.01
*R. erythropolis* SK121	84.7	98.22	0.018	0.07
*R. qingshengii* djl-6-2	88.8	98.73	0.0134	0.04
*R. qingshengii* MK1	90.0	98.82	0.0122	0.16
*R. qingshengii* CS98	89.7	98.8	0.0125	0.02
*R. qingshengii* TUHH-12	86.7	98.49	0.0158	0.65
*R. qingshengii* BKS 20-40	88.6	98.63	0.0136	0.03
*R. erythropolis* ACN1	54.2	93.97	0.0626	0.09
*R. erythropolis* AV96	62.0	95.49	0.0482	0.06
*R. erythropolis* 1159	62.2	95.51	0.0479	0.13
*R. erythropolis* VSD3	62.0	95.41	0.0482	0.02
*R. erythropolis* MI2	62.3	95.44	0.0477	0.13
*R. erythropolis* NSX2	62.3	95.52	0.0478	0.07
*R. erythropolis* NBRC 15567^T^	62.1	95.48	0.048	0.02
*R. erythropolis* R138	61.9	95.45	0.0484	0.08
*R. erythropolis* BG43	62.3	95.51	0.0478	0.1
*R. erythropolis* S-43	62.1	95.17	0.048	0.22
*R. erythropolis* DN1	63.0	95.57	0.0466	0.04
*R. erythropolis* CCM2595	62.7	95.54	0.0471	0.07
*R. erythropolis* XP	62.3	95.44	0.0477	0.08
*R. erythropolis* PR4	62.2	95.50	0.0479	0.07

**FIGURE 1:**
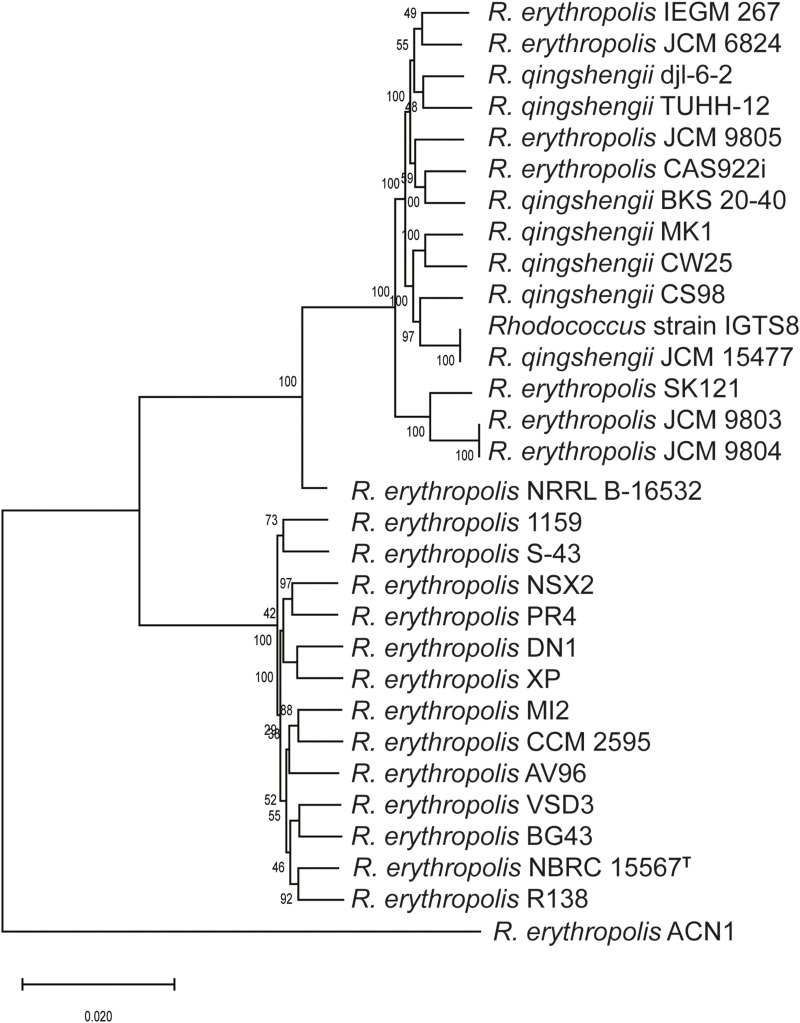
A maximum-likelihood phylogenomic tree generated from concatenated nucleotide sequences of the core genomes of the *R. erythropolis* and *R. qingshengii* strains. The scale bar represents the number of nucleotide substitutions per site.

The size and digital GC content of the genome of strain IGTS8 were similar to those of the strains received as *R. erythropolis* and *R. qingshengii*. These values ranged from 6.28 to 7.43 Mb and from 61.7 to 62.5 mol%, respectively (Table 1). A phylogenetic tree generated from concatenated core genome sequences of these strains showed that they fell into two distinct clades, one encompassing 13 *R. erythropolis* strains including *R. erythropolis* NBRC 15567^T^ and the other all of the *R. qingshengii* strains together with 7 strains received as *R. erythropolis* (Figure 1). Pairwise dDDH calculations indicated the presence of three species in the dataset (Supplementary Table S1). These results are consistent with previous studies in which *R. erythropolis* and *R. qingshengii* strains fell into a single taxon, namely species-group D (Sangal et al., 2016, 2019). Eight isolates initially classified as *R. erythropolis* (strains CAS922i, IEGM 267, JCM 6824, JCM 9803, JCM 9804, JCM 9805, NRRL B-16532 and SK121) showed ≥ 80% dDDH values with *R. qingshengii* JCM 15477^T^ and should be re-classified as *R. qingshengii*. Similarly, the dDDH values between *R. erythropolis* NBRC 15567^T^ and strains 1159, AV96, BG43, CCM 2595, DN1, MI2, NSX2, PR4, R138, S-43, XP and VSD3 varied from 85.7 to 90.2% which is consistent with their designation as *R. erythropolis*. One strain, *R. erythropolis* ACN1, formed a distinct phyletic line and probably merits species status (Figure 1 and Supplementary Table S1).

ANI values between members within the *R. qingshengii* and *R. erythropolis* clades varied between 97.41 and 99.86% (average: 98.43 ± 0.38; Supplementary Table S2) and 98.16–98.79% (average: 98.56 ± 0.13), respectively which is well above the cut-off point for assigning them to these species. Pairwise ANI values between the strains from the two clades were marginally higher than the 95–96% threshold suggesting that they might belong to a single species (ANI values: 95.11–96.43%; average: 95.53 ± 0.21). The corresponding AAI value between *R. erythropolis* NBRC 15567^T^ and *R. qingshengii* JCM 15477^T^ (AAI: 96.64%; SD: 7.14%) underpins this point.

**FIGURE 2:**
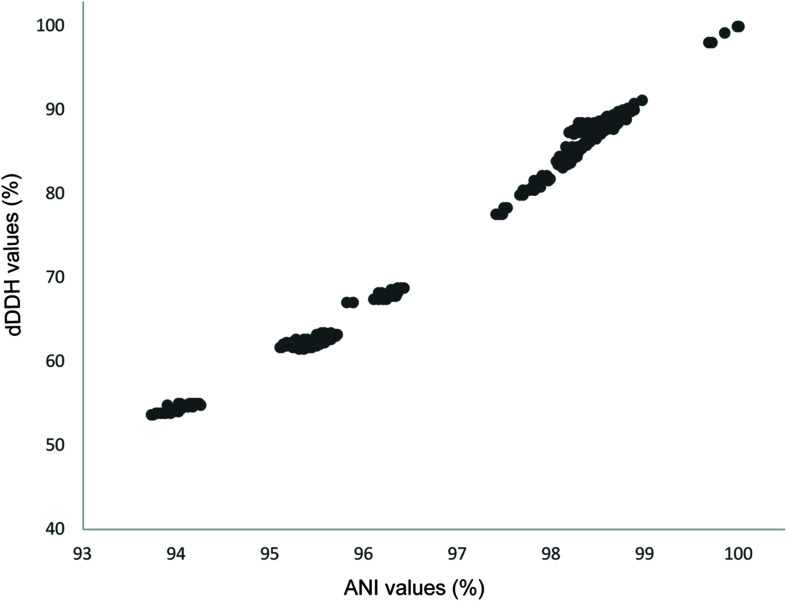
Correlation between dDDH and ANI values in the dataset.

*R. qingshengii* was proposed as a novel species based on phenotypic differences and an experimental DDH value of 23.8% between strain djl-6^T^ (JCM 15477^T^) and *R. erythropolis* DSM 43066^T^ (NBRC 15567^T^; Xu et al., 2007). Computational DDH values between the individuals of the two clades defined in the present study were 61.7–68.9% (average: 62.9 ± 1.4), values that are much higher than the corresponding experimental value between the *R. qingshengii* and *R. erythropolis* type strains but still below the 70% cut-off value (Supplementary Table S1). An ANI value of 96% was found to be suitable for *Aeromonas* species delineations that correlated with the dDDH cut-off of 70% (Colston et al., 2014). Similarly, in the present study ANI and dDDH values were found to be strongly correlated (*R*^2^ = 0.9932; Figure 2). It has been pointed out that the use of a universal cut-off point is not appropriate as ANI values reflect the methods used to determine them and that the evolutionary history of the taxa under study should be considered (Palmer et al., 2020).

A comparison of all 30 genomes revealed a pangenome encompassing 32,515 genes, including 1,985 core genes present amongst all of the strains (Supplementary Table S3). Based on the default protein BLAST identity of 95%, only 34 and 35 genes were found to be unique to strains in the *R. erythropolis* and *R. qingshengii* clades, respectively. However, 53–60% of these genes encode hypothetical proteins (Supplementary Table S3). For the remaining clade-specific genes, homologs (other genes encoding proteins with the same functions) were observed among the other clades (Supplementary Table S3). The default 95% identity cut-off is highly stringent for strains of different species which has potentially separated some of the orthologous genes as clade-specific genes.

Many genes (218) were found to be specific to strain IGTS8 with 116 encoding hypothetical proteins or proteins of unknown function and 10 belonging to mobile genetic elements such as transposases (Supplementary Table S3). Again, homologs of the remaining 92 genes with annotated functions were present among other strains. However, five genes encoding 3-hydroxy-3-isohexenylglutaryl-CoA:acetate lyase (re30.peg.6706), acetophenone carboxylase subunit Apc3 (re30.peg.6438), cyanate ABC transporter substrate binding protein (re30.peg.6484), a possible dicarboxylate carrier protein (re30.peg.6646) and a small heat shock protein (re30.peg.4427) appear to be specific to IGTS8 and may have introduced minor functional variations in strain IGTS8 in comparison to other *R. qingshengii* strains. Molecular investigations are required to understand the functional impact of these genes and those encoding hypothetical proteins on the strain IGTS8.

The genomic data from this study not only underline the close relationship between *R. erythropolis* and *R. qingshengii* strains (Xu et al., 2007; Dastager et al., 2014; Táncsics et al., 2014; Sangal et al., 2016, 2019) but also show that the case for assigning them to a single species is problematic. In such instances, recommended thresholds of genomic indices for species delineation not only need to be interpreted with care but should also take into account the broader taxonomic properties of the organisms (Colston et al., 2014; Riesco et al., 2018). Given the assignment of the IGTS8 strain to a distinct phylogenomic clade corresponding to *R. qingshengii* (Figure 1), supporting *in silico* dDDH similarity values (Table 3) and genomic features (Supplementary Tables S1, S2) together with available phenotypic data (Gray et al., 1996; Xu et al., 2007; Jones and Goodfellow, 2012), it is prudent to continue to recognize the taxonomic status of *R. erythropolis* and *R. qingshengii*.

### Biosynthetic Potential

The number of predicted biosynthetic gene clusters (BGCs) was similar amongst the *R. erythropolis* and *R. qingshengii* strains ranging between 13 and 24 (Supplementary Table S4). The draft assemblies with higher numbers of contigs are predicted to have more gene clusters than the finished genomes, e.g., *R. erythropolis* strain S-43 with 533 contigs has the highest number of gene clusters. The six complete genomes of strains assigned to the two species have 15–17 BGCs (Figure 1; Supplementary Table S4). These observations are consistent with those of Sanchez-Hidalgo et al. (2018) who identified more gene clusters in genomes with smaller contigs amongst *Amycolatopsis* strains. The BGCs were assigned to 12 types all of which were detected in the genomes of both the *R. erythropolis* and *R. qingshengii* strains. In contrast, Ceniceros et al. (2017) found that predicted BGCs in rhodococci, including *R. erythropolis* strains, were clade-specific and lacked any homology with gene clusters encoding known natural products.

AntiSMASH predicts BGCs and potential products based on the percentage of genes from the closest known BGC showing significant BLAST hits to the query genome (Blin et al., 2019). In this study, twelve gene clusters including four BGCs potentially encoding bacteriocin, ectoine, heterobactin A/heterobactin S2 (non-ribosomal peptide synthetase, NRPS) and erythrochelin (NRPS) were highly conserved among the *R. erythropolis* or *R. qingshengii* strains and were predicted among 21–30 genomes (Supplementary Table S4). The cluster potentially encoding bacteriocin was present in all 30 strains with 75–100% of genes similar to known bacteriocin-producing clusters (Supplementary Table S4). Bacteriocins are commonly produced by bacteria to inhibit other strains competing for resources, some are extensively used in the food industry (Riley and Wertz, 2002; Settanni and Corsetti, 2008; Yang et al., 2014). The BGC predicted to encode ectoine biosynthesis was also identified in all 30 strains. Ectoine, a protective molecule, enables bacteria to survive in extreme conditions (Graf et al., 2008), indicating that *R. erythropolis* and *R. qingshengii* strains may be able to withstand harsh environmental conditions such as osmotic stress. In addition, it is used commercially as an osmotic stabilizing agent in healthcare and cosmetics (Kunte et al., 2014; Czech et al., 2018).

The number of NRPS clusters ranged from 5 out of 15 in *R. erythropolis* strain PR4 to 16 out of 24 in *R. erythropolis* strain VSD3 (Supplementary Table S4). Five NRPS gene clusters were highly conserved in the dataset, including a cluster with 90–100% of the genes coding for heterobactin A/heterobactin S2 biosynthesis (Supplementary Table S4). Only *R. erythropolis* strains NBRC 15567^T^ and S-43 showed lower proportions of genes (36 and 63%, respectively) similar to those coding for heterobactin A/heterobactin S2 biosynthetic enzymes. Heterobactins are siderophores involved in iron uptake which is essential for bacterial growth. Heterobactin biosynthesis was previously detected in *R. erythropolis* PR4 and the IGTS8 strains (Carran et al., 2001; Bosello et al., 2013). A predicted erythrochelin BGC was found to be conserved among 27 genomes (Supplementary Table S4). However, the corresponding cluster in *R. qingshengii* strain JCM 15477^T^ was shown to encompass a relatively lower proportion (42%) of genes when compared to known erythrochelin BGCs. Erythrochelin is another class of siderophores involved in iron uptake (Robbel et al., 2010). A third NRPS cluster showed partial gene similarities (18–27% of the genes) to that encoding coelichelin biosynthesis, a siderophore reported in *Streptomyces coelicolor* (Challis and Ravel, 2000). Siderophores have a wide range of roles as biosensors and as bioremediation and chelation agents involved in weathering of soil minerals and in enhancing plant growth (Ahmed and Holmstrom, 2014).

One of the conserved NRPS biosynthetic clusters present in the genomes of 22 of the strains showed limited gene similarities (3–4% of the genes) to those encoding rifamorpholines A-E (Supplementary Table S4). Rifamorpholines A-E are macrolactams, a new subclass of rifamycin antibiotics produced by *Amycolatopsis* strain HCa4 which show antimicrobial activity against methicillin-resistant *Staphylococcus aureus* (Xiao et al., 2017). A NRPS-terpene hybrid cluster was detected in the genomes of 27 strains with 6% of genes similar to a BGC responsible for biosynthesis of SF2575 (an anticancer tetracycline; Pickens et al., 2009).

Conserved BGCs include a butyrolactone and a lanthipeptide cluster (no known similarities to any existing BGC), a cluster for biosynthesis of a linear azol(in)e-containing peptide (LAP) type (11% of the genes similar to those encoding diisonitrile antibiotic SF2768), a terpene biosynthesis cluster (18–42% of the genes similar to ones encoding an isorenieratene pigment) and a type 1 polyketide synthase (T1PKS; 6–8% similar to a kirromycin-encoding cluster). Butyrolactones are involved in intracellular signaling and are intermediates in the biosynthesis of other molecules that have an important role in metabolism and stress response (Du et al., 2011). Lanthipeptides, also known as RiPPs (ribosomally synthesized and post-translationally modified peptides) are involved in multiple biological activities, including inhibition of pathogenic bacteria (Repka et al., 2017; Ongey et al., 2018). Diisonitrile SF2768 is responsible for chelation and transport of copper and has antifungal properties (Wang et al., 2017; Xu and Tan, 2019) while polyketides are known as antimicrobial, immune-suppressing and antifungal agents (Gomes et al., 2013).

In addition to the conserved BGCs, other clusters were present in some of the *R. erythropolis* and *R. qingshengii* strains (Supplementary Table S4). For instance, the genomes of *R. qingshengii* strains JCM 6824, JCM 15477 and IGTS8 contained a cluster showing 81% similarity to genes encoding arylpolyene (aurachin RE) and a cluster in the genome of *R. erythropolis* strain JCM 9804 had 60% of the genes similar to those encoding albachelin biosynthesis. Arylpolyenes are bacterial pigments structurally and functionally similar to carotenoids (Schoner et al., 2016) and are active as antimicrobial and antioxidant agents (Narsing Rao et al., 2017). Albachelin is a siderophore that was initially isolated from *Amycolatopsis alba* (Kodani et al., 2015). Other BGCs showed either no or limited similarities to known clusters and are likely to produce novel metabolites. Similarly, other NRPS BGCs showed partial similarities to different antibiotic-producing clusters (Supplementary Table S4). These clusters appear quite diverse compared with previously characterized clusters and may encode novel antibiotics and hence need molecular characterization.

In total, 17 BGCs were detected in strain IGTS8 (Supplementary Table S4). According to ClusterBLAST results, 15 BGCs potentially encoding biosynthesis of 9−methylstreptimidone (NRPS), bacillomycin D (NRPS), bacteriocin, butyrolactone, coelichelin (NRPS), ectoine, erythrochelin (NRPS), heterobactin A/heterobactin S2 (NRPS), isorenieratene (terpene), kirromycin (T1PKS), monensin (NRPS) and rifamorpholine A-E were also present among other *R. erythropolis*, *R. qingshengii* and some undefined *Rhodococcus* strains (Supplementary Figure S1: BGCs 2–11 and 13–17). The BGC predicted to encode aurachin RE biosynthesis was distantly related (3–5% gene similarity) to multiple *Streptomyces* strains including *S. rosechromogenus* (5%), and to strains of *Amycolatopsis nigriscens* (5%), *Micromonospora tulbaghiae* (3%) and *Saccharothrix espanaenesis* (4%; Supplementary Figure S1: BGC 12). This BGC was also present in two additional strains in the *R. qingshengii* clade (Supplementary Table S4). A BGC encoding a linear azol(in)e-containing peptide (similar to diisonitrile antibiotic SF2768) showed high relatedness to *Frankia* strain BMG 5.23 (75% of genes in the BGC) and moderate similarity (17–47% of genes in the BGC) with other *Frankia* strains, and with strains of *Williamsia muralis*, *Rhodococcus fascians*, and *Kibdelospoangium phytohabitans* (Supplementary Figure S1: BGC 1). Overall, these results demonstrate the presence of multiple BGCs in the dataset that are potentially capable of producing novel compounds and are largely shared by *R. erythropolis* and *R. qingshengii* strains.

### Biodesulfurization Genes-the *dsz* Operon

*R. qingshengii* strain IGTS8 is the prototype of fuel-biodesulfurizing bacteria harboring the 4S biodesulfurization pathway which allows the use of diesel-born organosulfur compounds, such as dibenzothiophene as a sole sulfur source, converting it to 2-hydroxybiphenyl and releasing the sulfur atom as sulfite (Oldfield et al., 1998). The 4S pathway enzymes are encoded by the plasmid-borne *dsz* operon (Denome et al., 1994; Santos et al., 2007; Khan et al., 2017). A search for this *dsz* operon (Piddington et al., 1995) amongst the *R. qingshengii* and *R. erythropolis* strains showed that it was only present in the genome of *R. erythropolis* XP. This soil isolate, which possesses a plasmid carrying the *dsz* operon, is able to desulfurize benzonaphthothiophene (Yu et al., 2006; Tao et al., 2011). Interestingly, *R. qingshengii* IGTS8 and *R. erythropolis* XP strains belong to different rhodococcal clades (Figure 1) suggesting that the plasmid carrying the *dsz* operon may be transferable between *R. qingshengii* and *R. erythropolis* strains via horizontal gene transfer. This proposition is in line with the fact that the *dsz* genes are usually carried on conjugative plasmids close to transposable elements, such as insertion sequences (Kayser et al., 2002; Kilbane and Le Borgne, 2004; Mohebali and Ball, 2016).

## Conclusion

The phylogenomic analyses show that *R. erythropolis* and *R. qingshengii* strains are members of very closely related species that belong to the *R. erythropolis* species-group. They also show that the catabolically active organism *Rhodococcus* strain IGTS8 is a *bona fide* member of *R. qingshengii*. Strains assigned to each of these species were shown to have genomes that contained 12 highly conserved BGCs, most of which showed limited similarity with clusters coding for the biosynthesis of known specialized metabolites thereby providing further evidence that rhodococci should feature prominently in drug discovery programmes. The finding that the genome of *R. erythropolis* strain XP, like that of *R. qingshengii* strain IGTS8, contains the biodesulfurization *dsz* operon suggests that plasmids carrying this operon may be readily transferable between *R. erythropolis* and *R. qingshengii* strains.

## Data Availability Statement

The datasets presented in this study can be found in online repositories. The names of the repository/repositories and accession number(s) can be found in the article/Supplementary Material.

## Author Contributions

WI and HM conceived the study. VS designed the strategy for the data analyses. VC and SK extracted genomic DNA, performed sequencing and assembled the reads into scaffolds. DT and VS analyzed the data. MG helped with the taxogenomic analyses and classification of strain IGTS8. HM, DH, CC, and AV provided intellectual input on the biosynthetic and catabolic potential of the bacterial strains. VS, WI, and MG drafted the manuscript. All authors contributed to the article and approved the submitted version.

## Conflict of Interest

The authors declare that the research was conducted in the absence of any commercial or financial relationships that could be construed as a potential conflict of interest.

## Footnotes

1 https://www.ncbi.nlm.nih.gov/tools/vecscreen/about/

2 https://github.com/djhshih/pairfq

3 https://www.bioinformatics.babraham.ac.uk/projects/fastqc/

4 http://enve-omics.ce.gatech.edu/aai/
