# Supplementary material for: Phylogenomic Classification and Biosynthetic Potential of the Fossil Fuel-Biodesulfurizing Rhodococcus Strain IGTS8
Source: Front Microbiol. 2020 Jul 7;11:1417. doi: 10.3389/fmicb.2020.01417 (PMC7358434; doi:10.3389/fmicb.2020.01417)

Supplementary Figure S1. ClusterBLAST output for 17 BGCs predicted in strain IGTS8 showing relatedness with closest genomes

BGC 1: LAP diisonitrile antibiotic SF2768

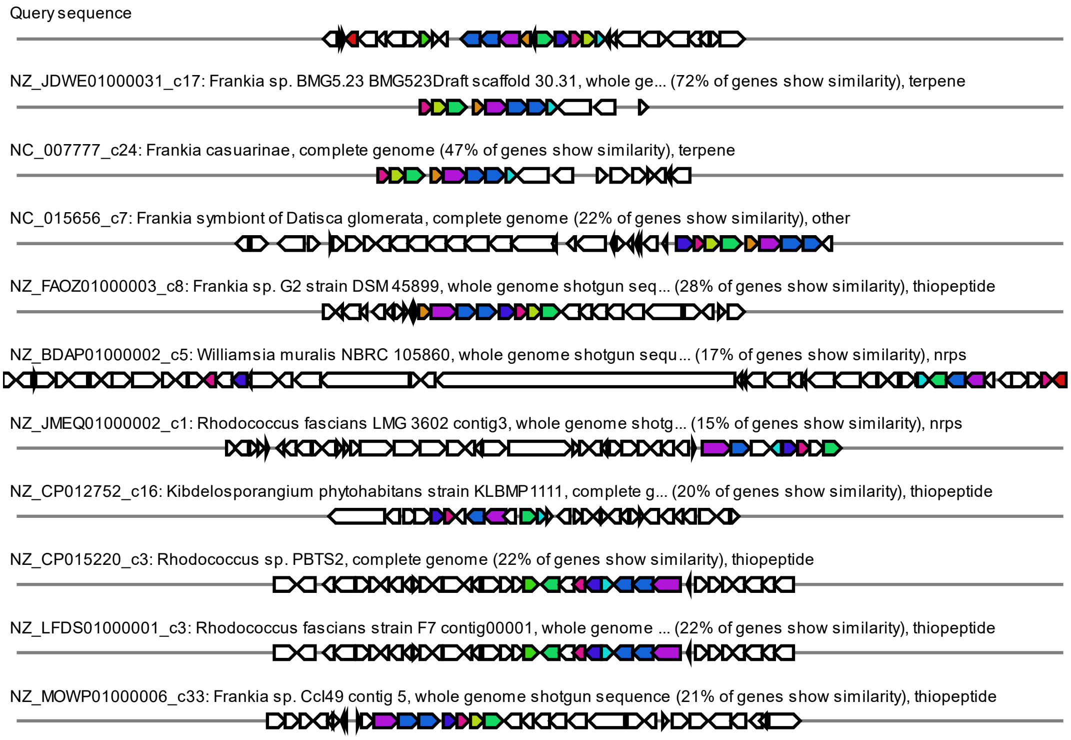

BGC 2: T1PKS

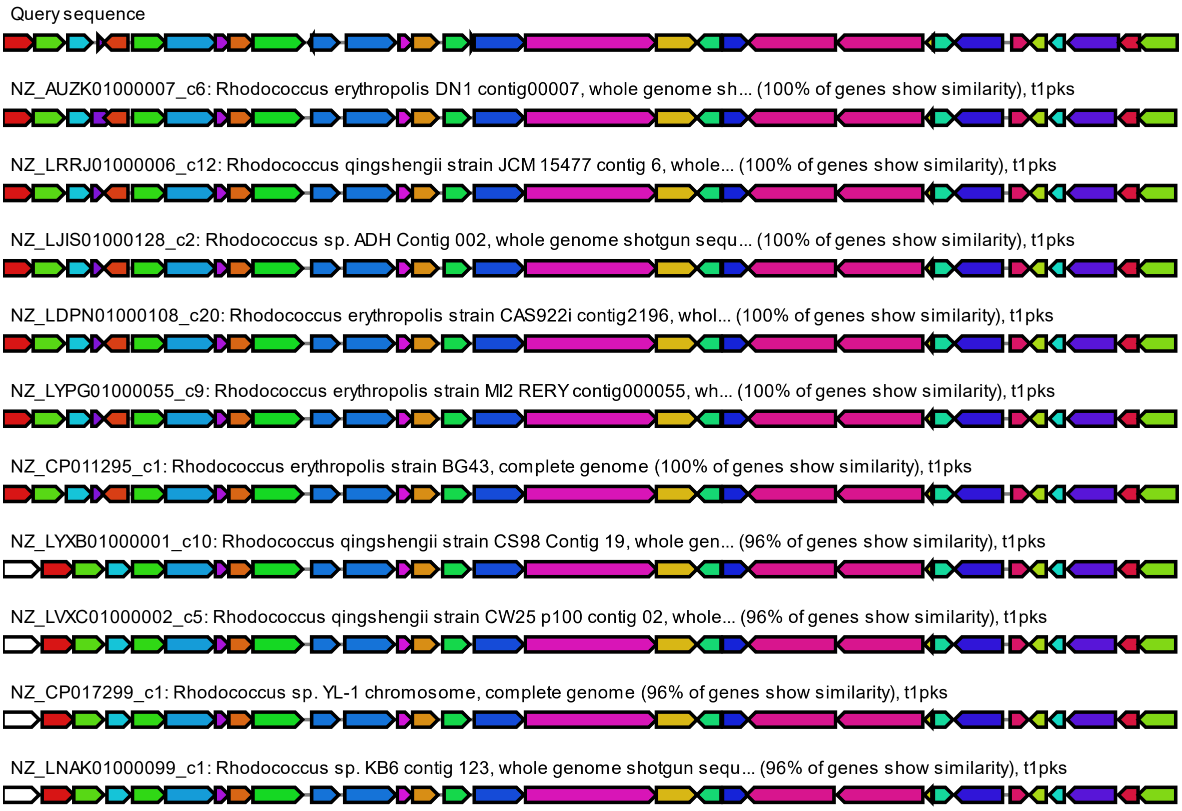

BGC 3: T1PKS kirromycin

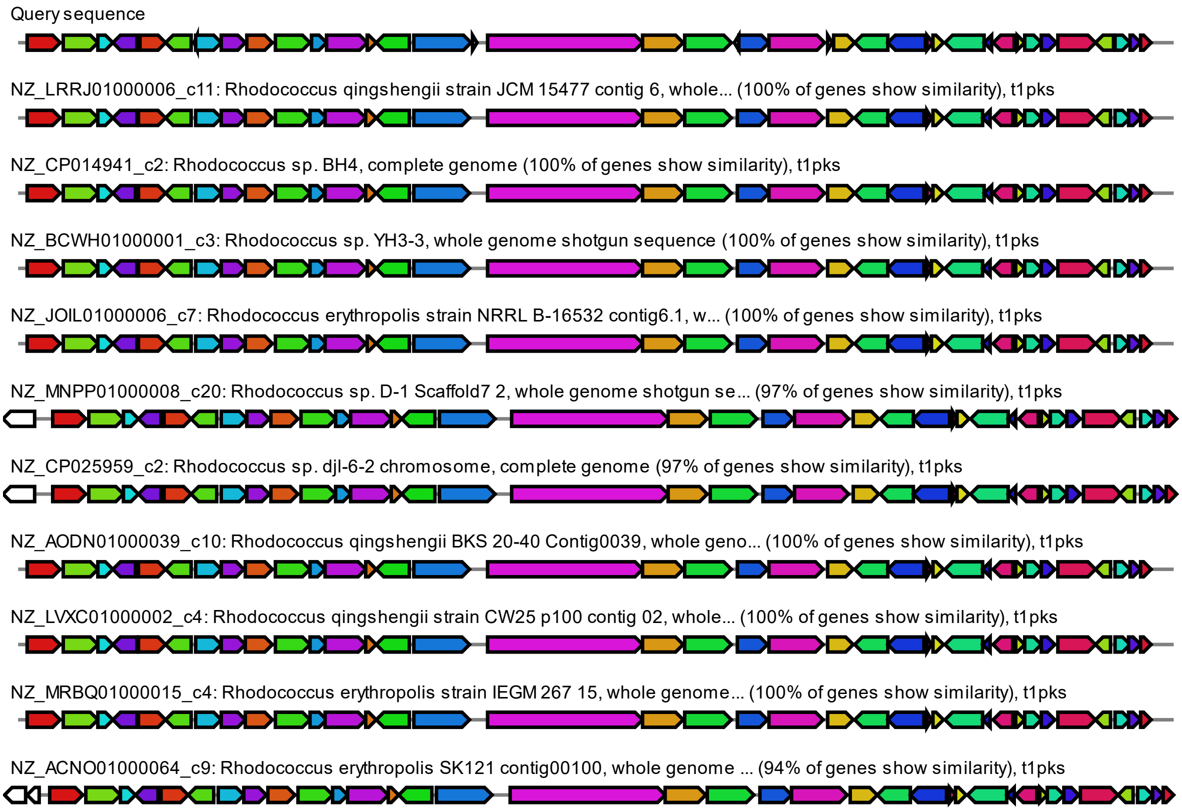

BGC 4: NRPS erythrochelin

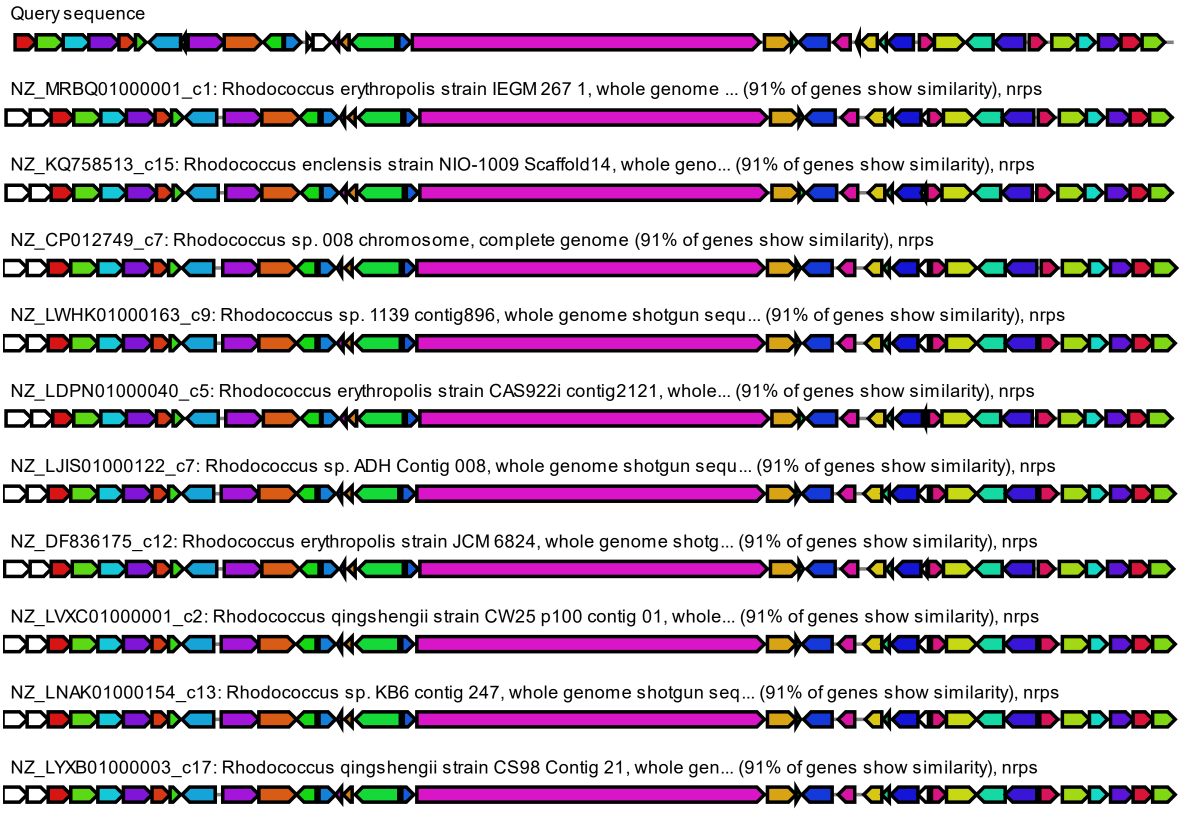

BGC 5: NRPS bacillomycin D

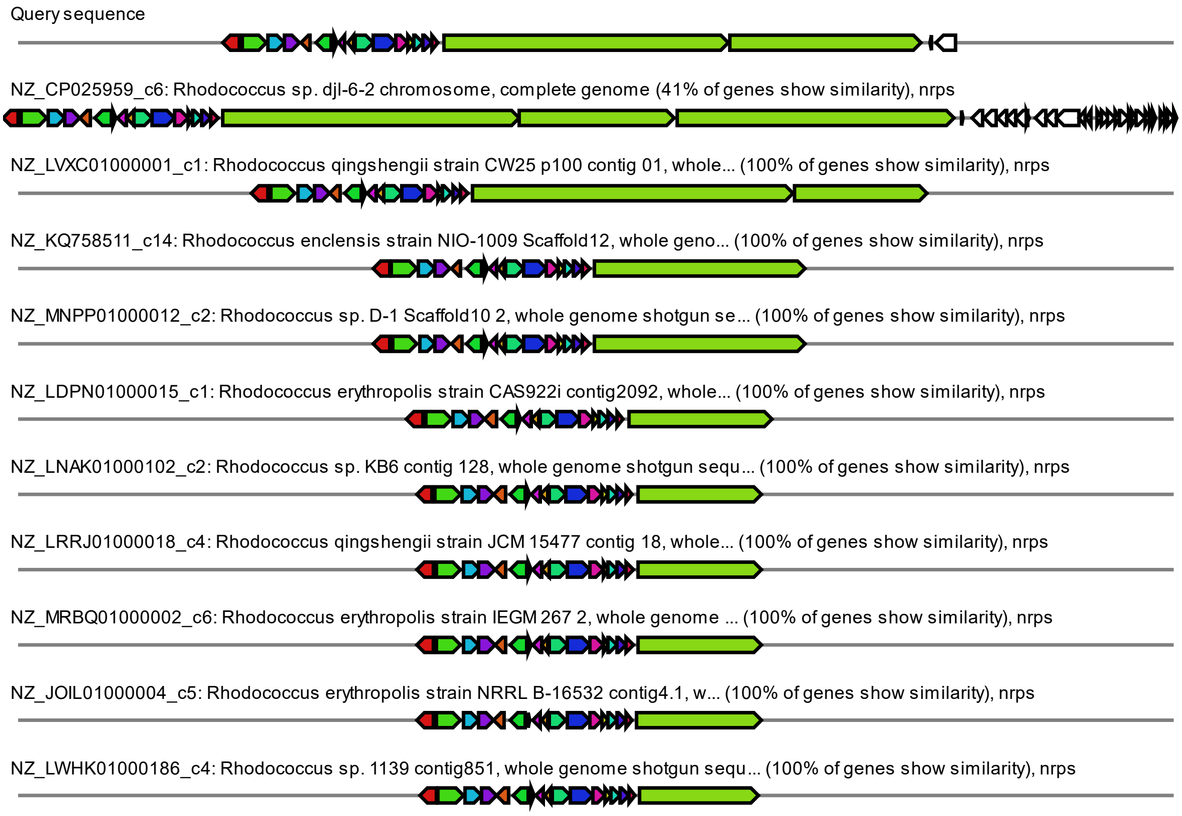

BGC 6: NRPS heterobactin A / heterobactin S2

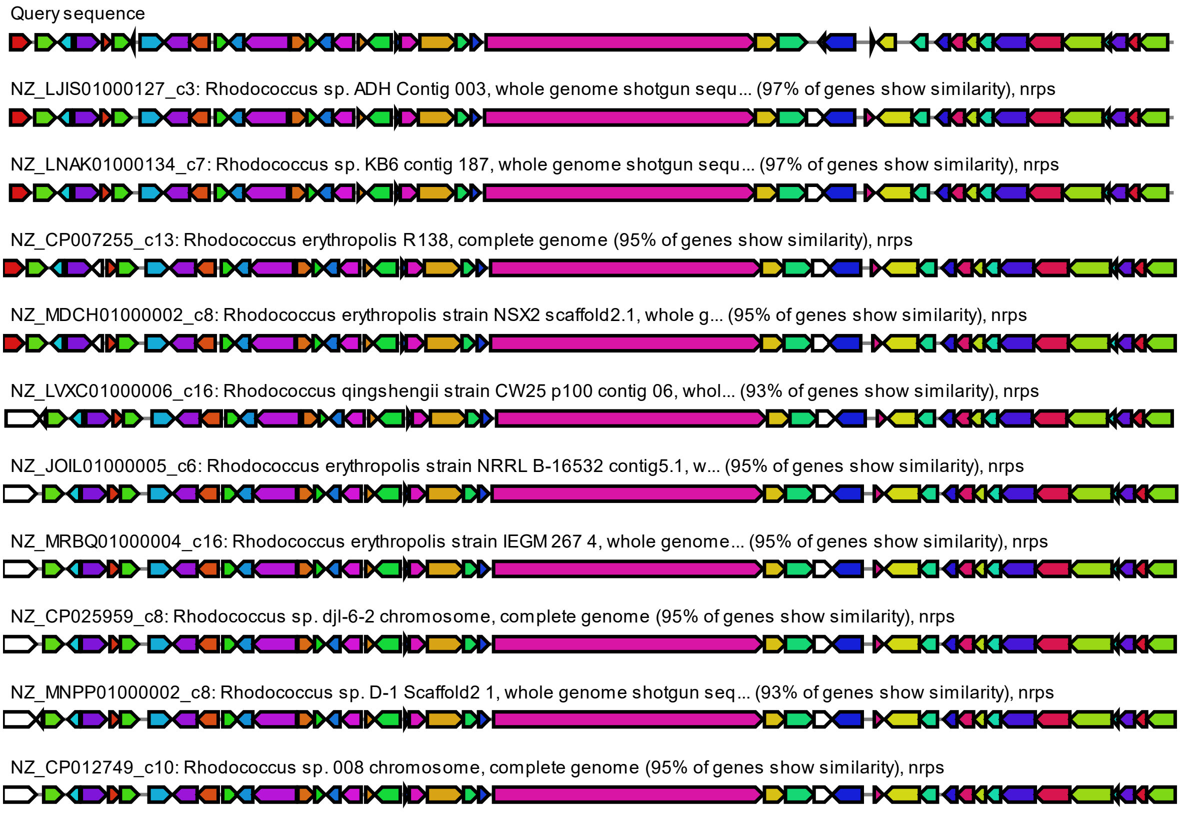

BGC 7: NRPS, terpene SF2575

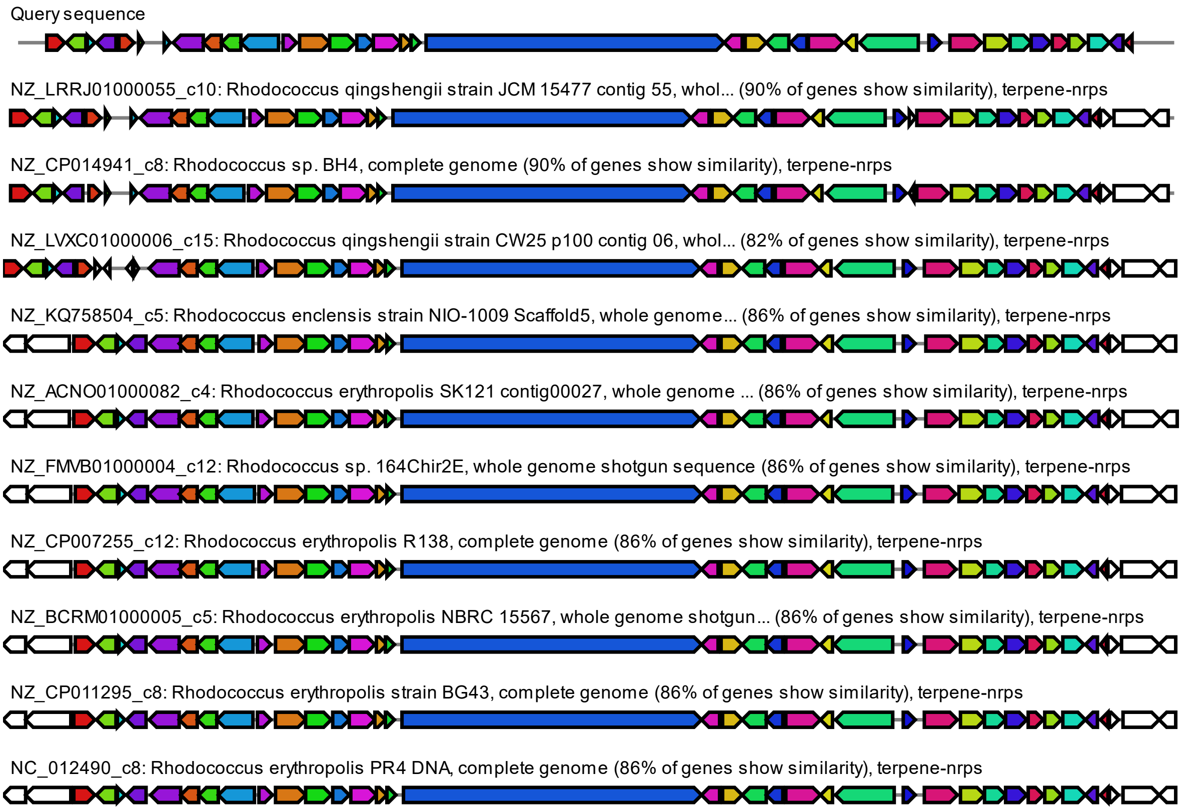

BGC 8: NRPS coelichelin

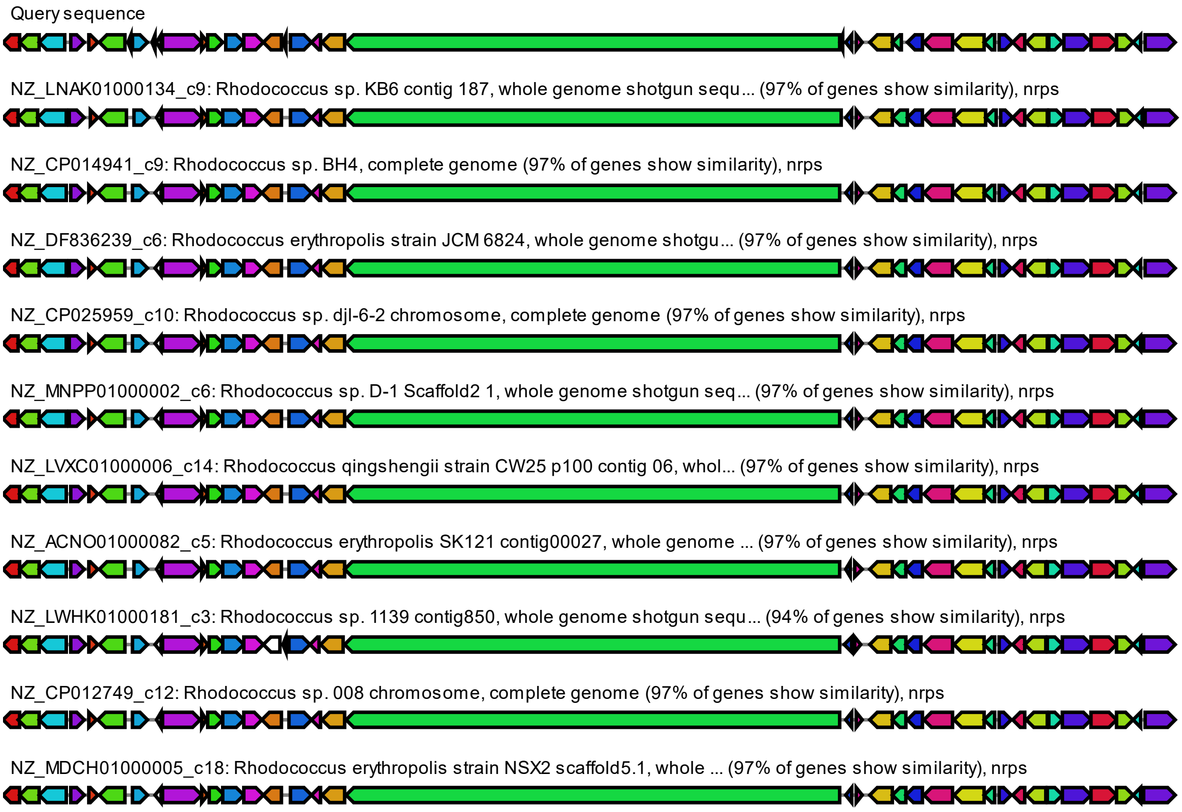

BGC 9: NRPS rifamorpholine A / rifamorpholine B / rifamorpholine C / rifamorpholine D / rifamorpholine E

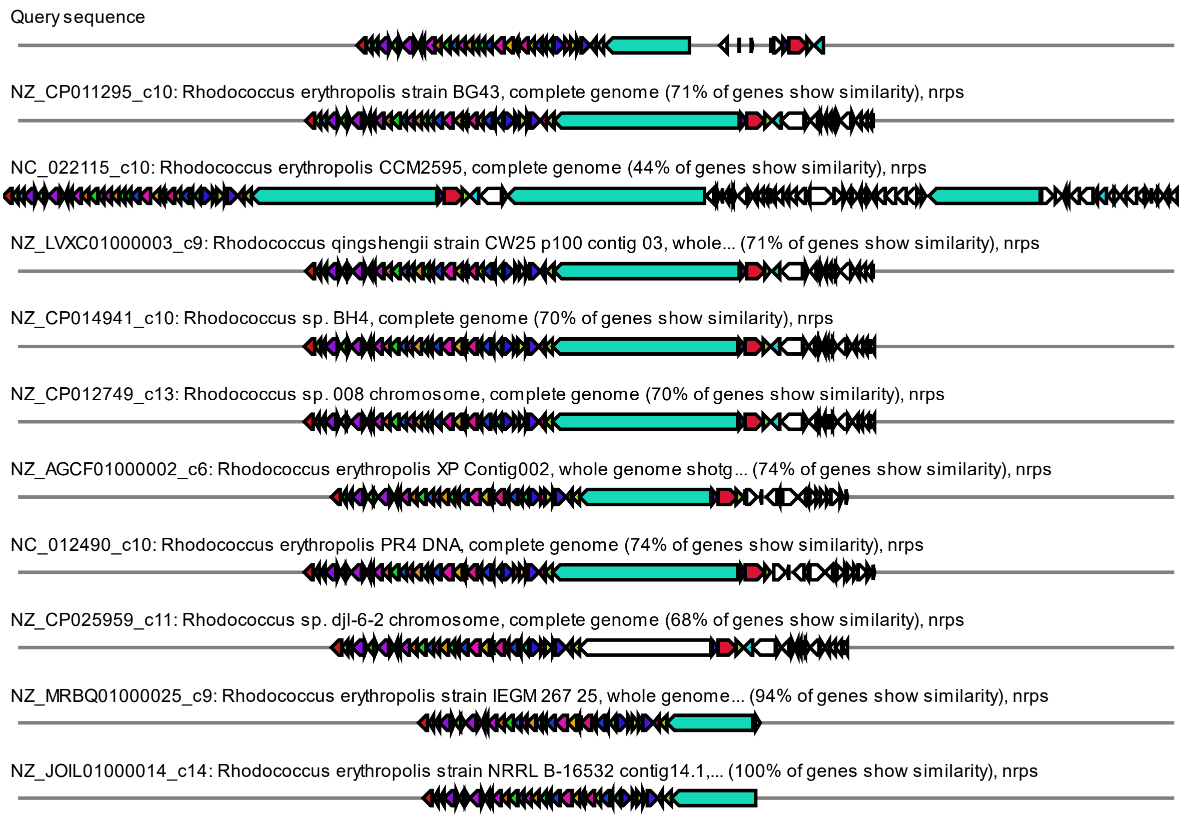

BGC 10: NRPS monensin

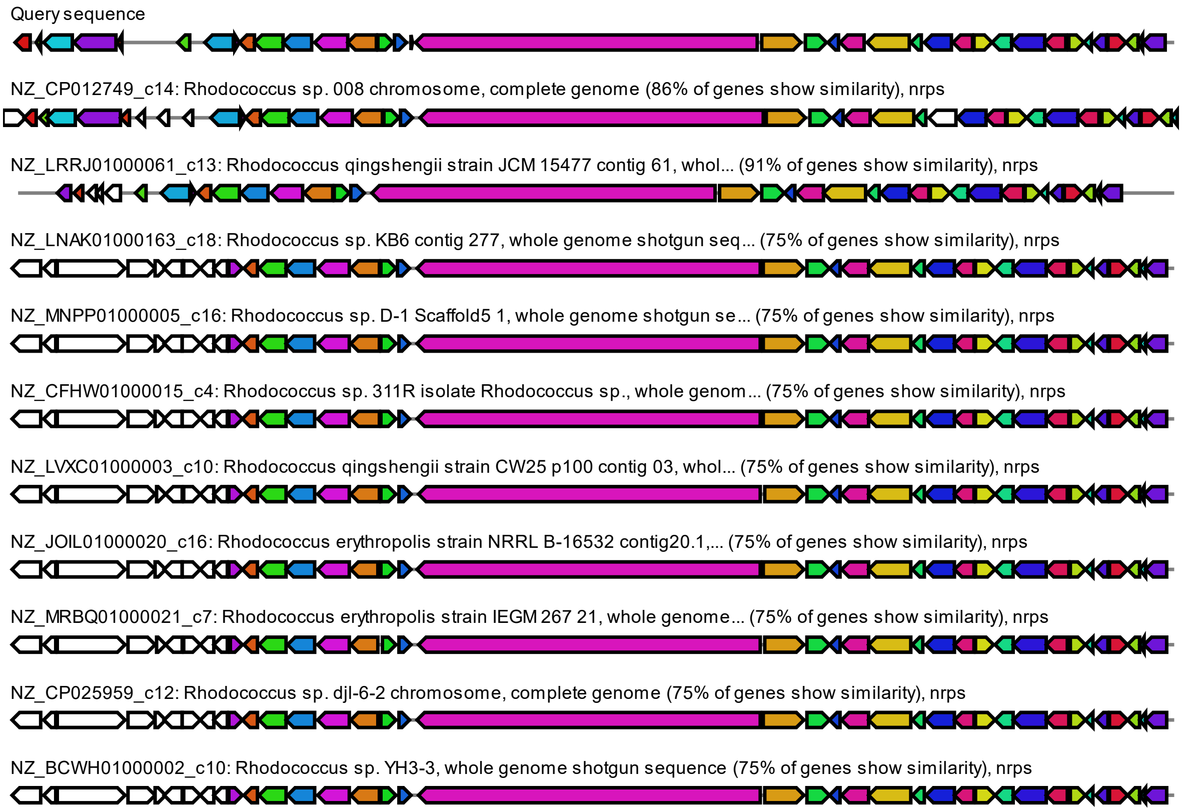

BGC 11: terpene isorenieratene

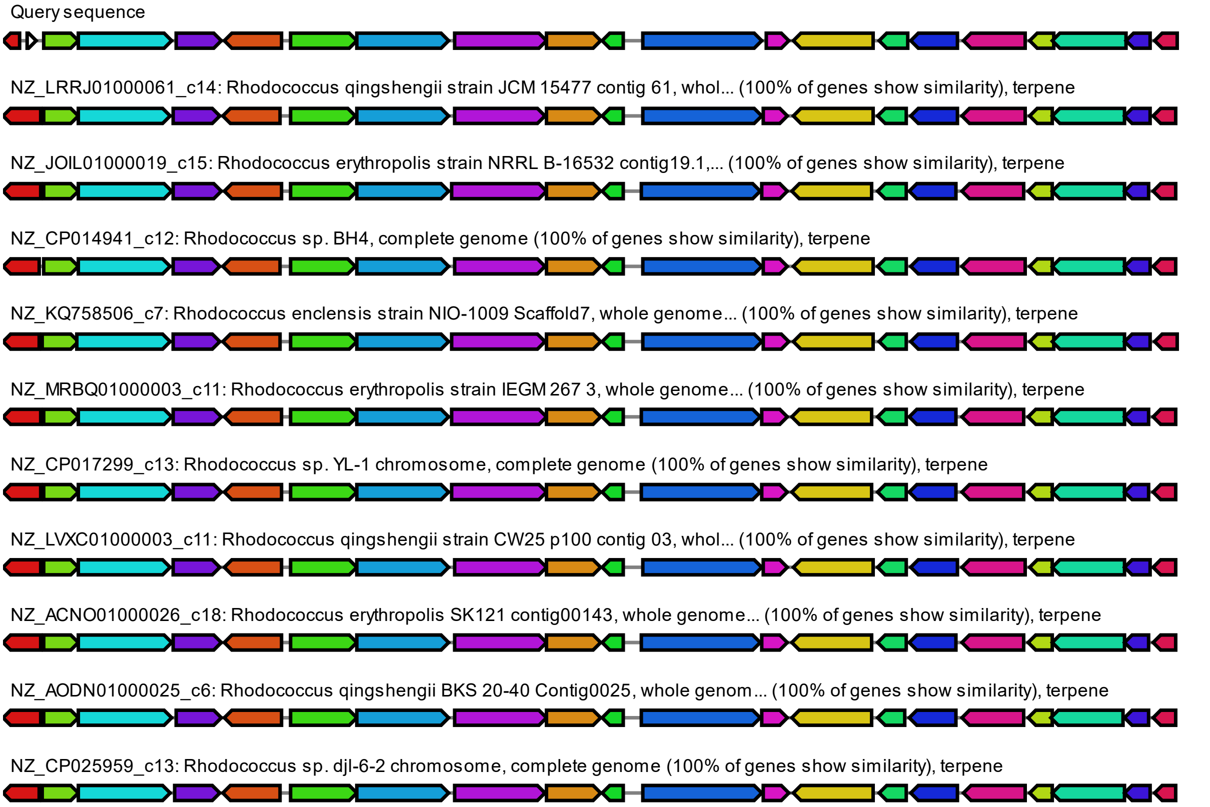

BGC 12: arylpolyene aurachin RE

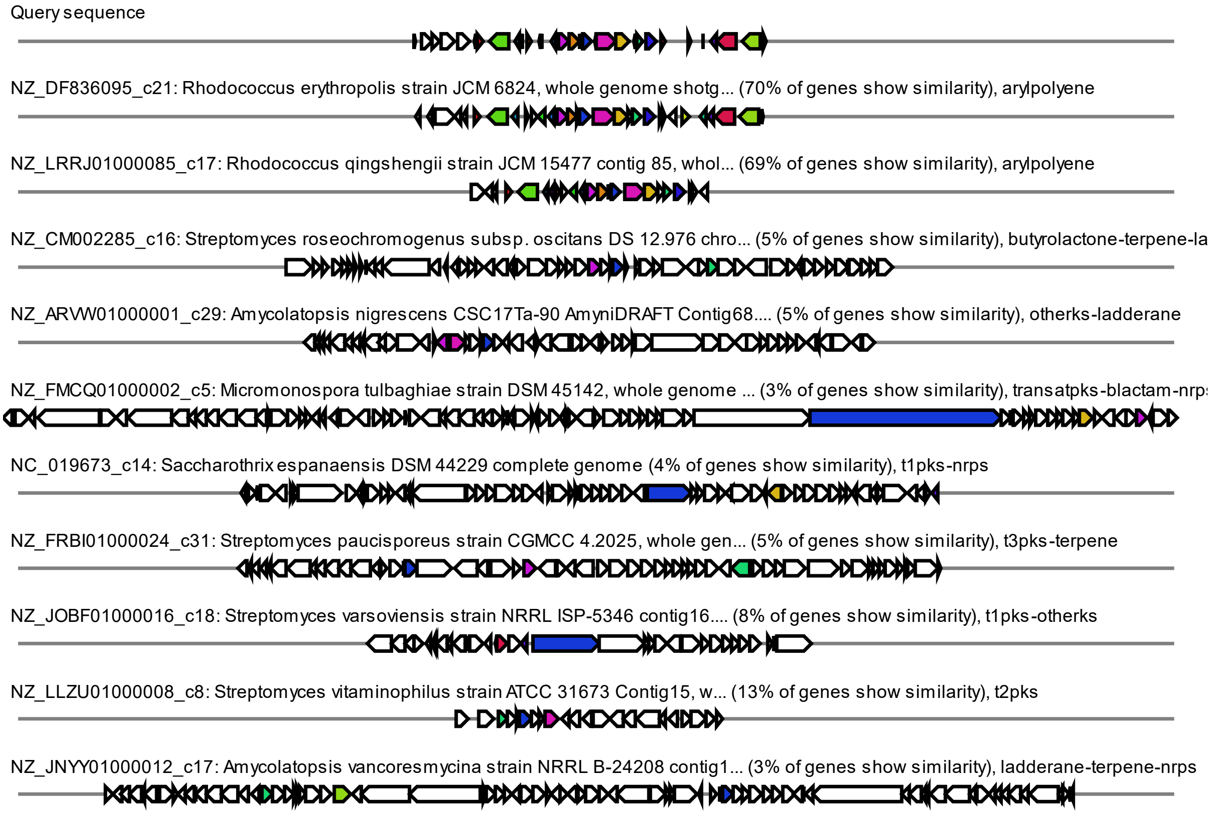

BGC 13: ectoine

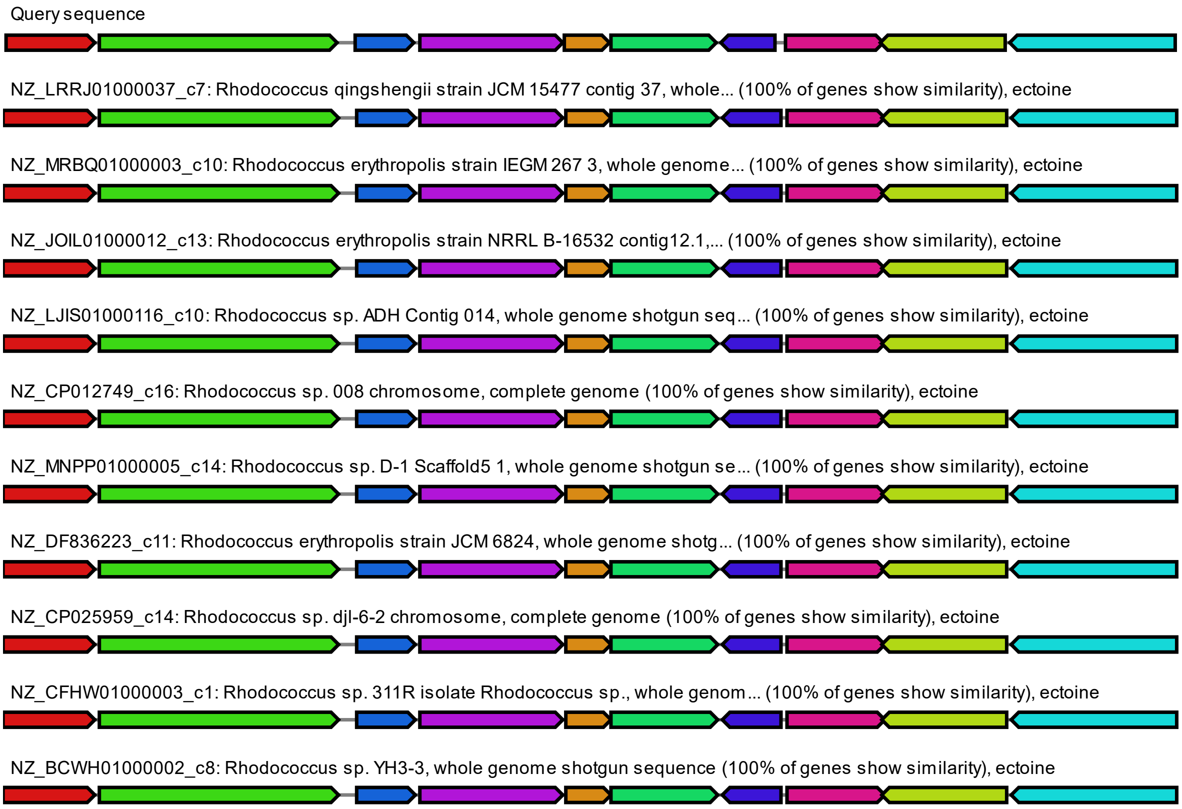

BGC 14: butyrolactone

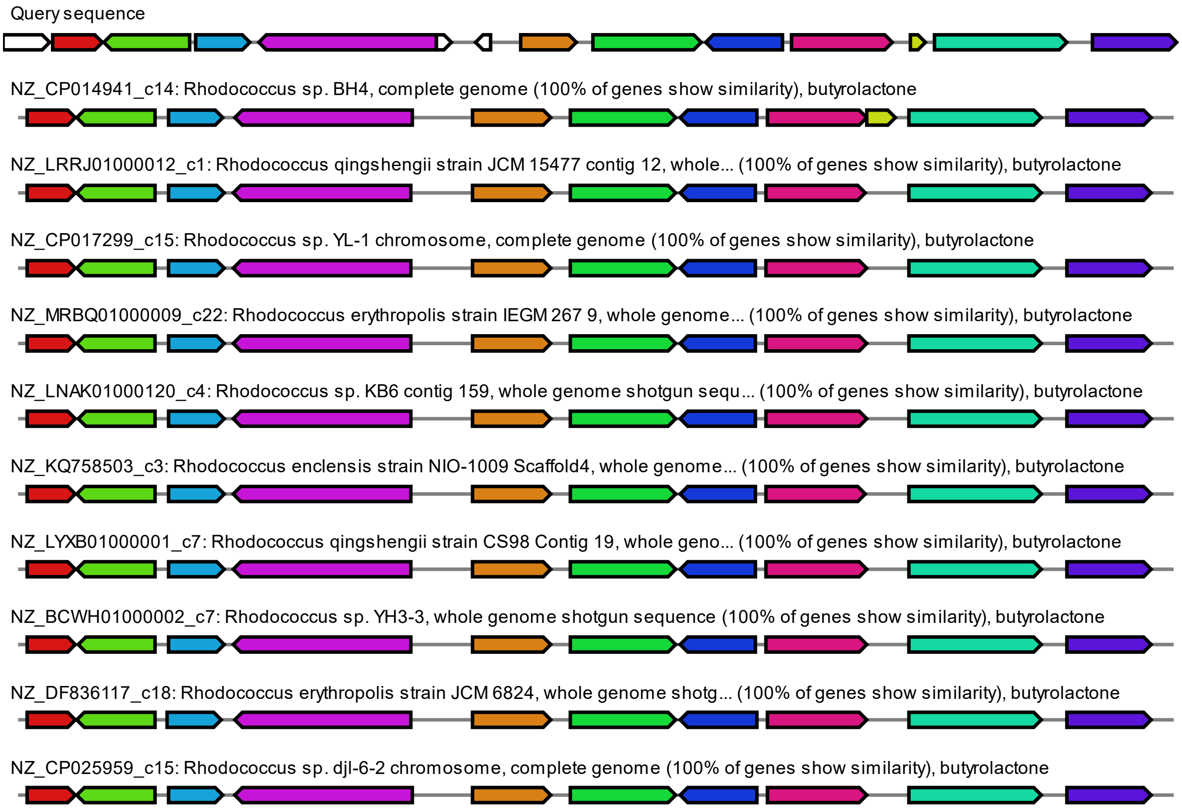

BGC 15: lanthipeptide cluster

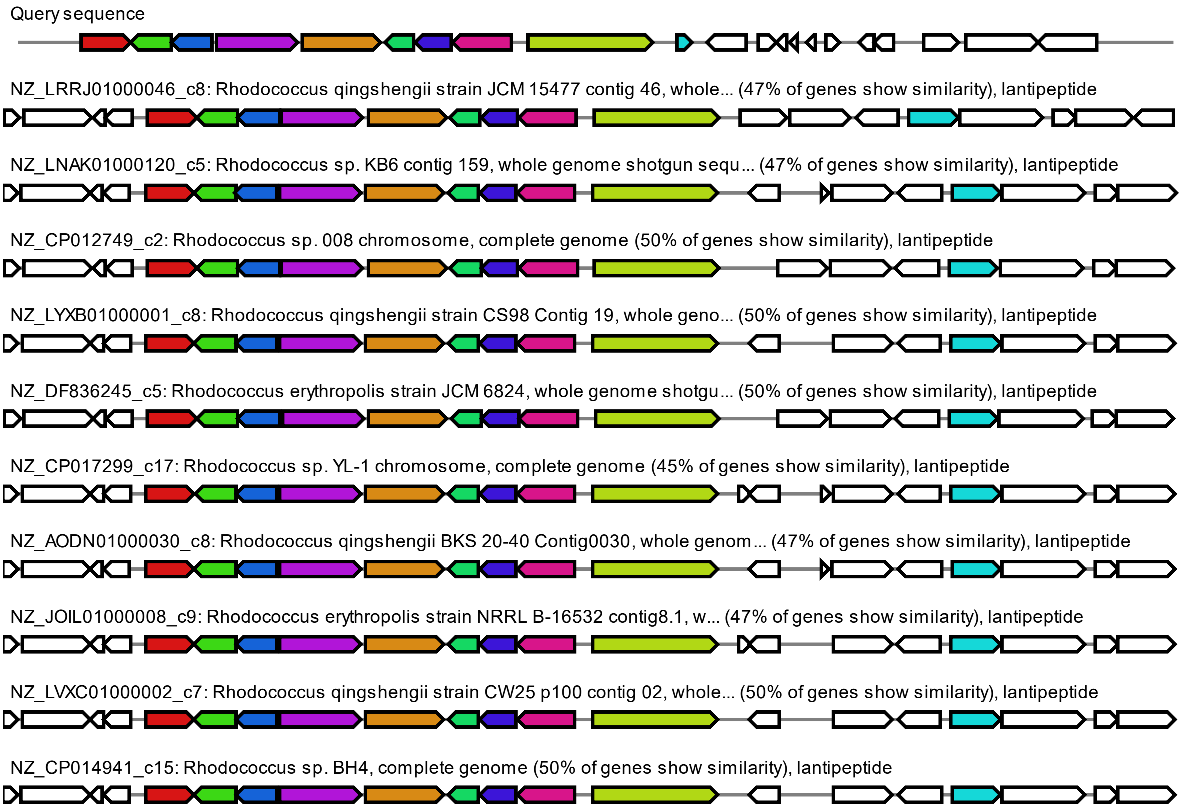

BGC 16: NRPS 9-methylstreptimidone

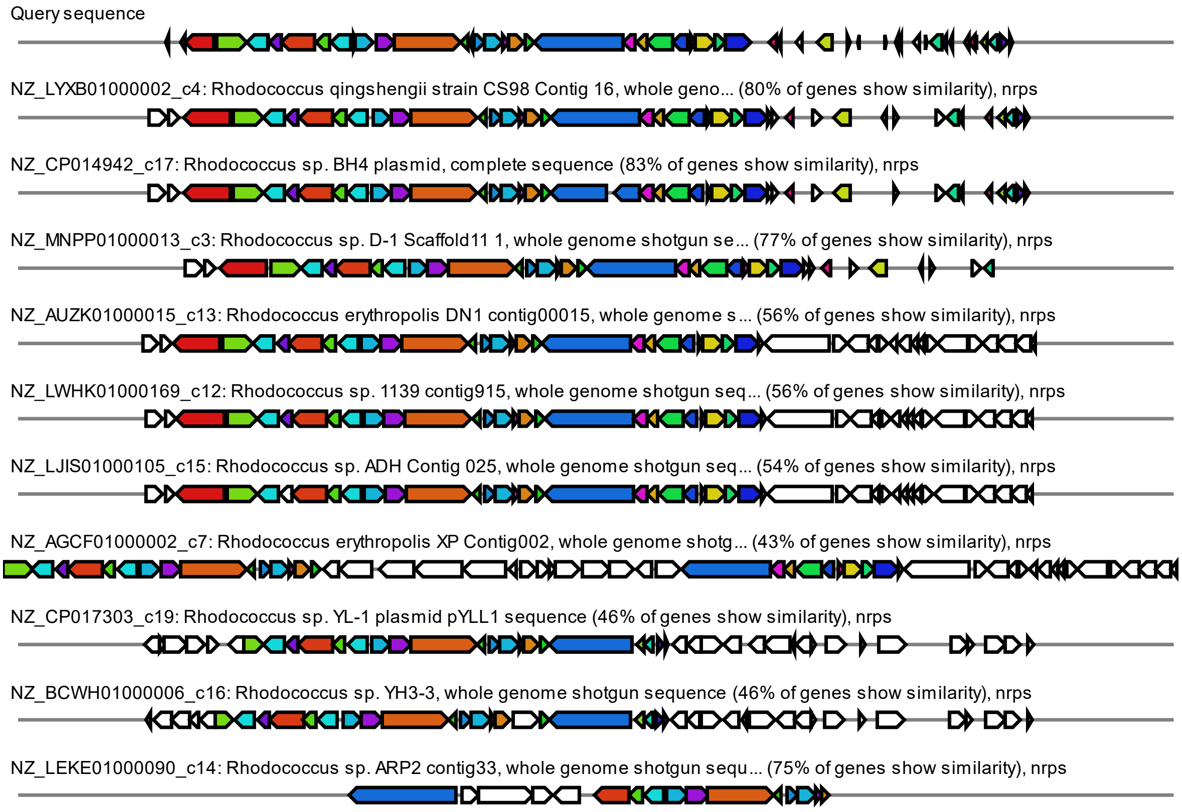

BGC 17: bacteriocin branched-chain fatty acids

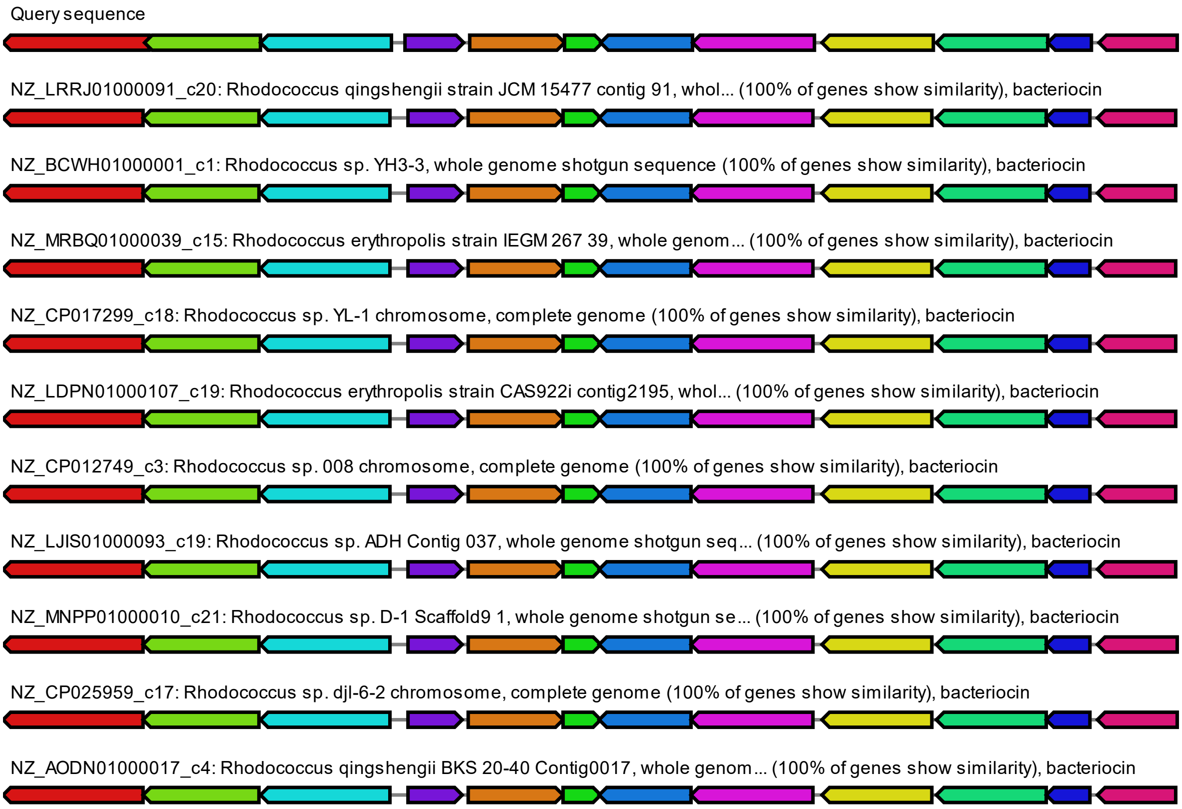

Supplement: Supplementary file 1 [file Image_1.pdf]
